# LMS-ViT: a multi-scale vision transformer approach for real-time smartphone-based skin cancer detection

**DOI:** 10.3389/frai.2025.1612502

**Published:** 2025-09-03

**Authors:** A. Anny Leema, P. Balakrishnan, G. Gopichand, G. Rajarajan

**Affiliations:** School of Computer Science and Engineering, Vellore Institute of Technology, Vellore, India

**Keywords:** vision transformer, domain adaptation, CNN, image classification, LMS-ViT

## Abstract

Skin cancer is the abnormal growth of skin cells. It occurs mostly in skin exposed to sunlight. To prevent the occurrence of skin cancer, avoid exposing skin to ultraviolet radiation. Skin cancer can be very harmful if found very late. Traditional convolutional neural networks (CNNs) face challenges in fine-grained lesion classification due to their limited ability to extract detailed features. To overcome such limitations, we introduced a novel approach in the form of a lightweight multi-scale vision transformer (LMS-ViT) application for the automated detection of skin cancer using dermoscopic images and the HAM10000 dataset. Unlike CNNs, LMS-ViT employs a multi-scale attention mechanism to capture both global lesion structures and fine-grained textural details, improving classification accuracy. This study combines skin images from the HAM10000 dataset with pictures taken using a smartphone. It uses a compact method to mix important features, which makes the system faster and suitable for real-time use in medical apps. The proposed system enables real-time skin cancer classification via a smartphone camera, making it portable and platform-independent. Experimental results show that LMS-ViT surpasses CNN-based models across all skin lesion categories, achieving 90% accuracy, an 18% improvement over CNN, while reducing computational cost by 30%. LMS-ViT also improves precision, recall, and F1-score, particularly in complex categories such as Vasc (0.96 to 1.01) and Nv (0.94 to 1.01), demonstrating superior classification power. With real-time android implementation, LMS-ViT offers accessible, mobile-friendly diagnostics for early skin cancer detection.

## 1 Introduction

Skin cancer develops majorly on areas of skin exposed to the sun. This includes the scalp, head, arms, face, and legs in women. However, skin cancer can also form on areas that are not exposed to the sun, for example your palm, toenails or fingernails, and the genital area. It can also affect people with different skin tones, for example, this type of cancer can also affect people with dark complexions. If a person with a dark complexion is affected by some skin cancers such as melanoma, then it is more likely that the areas not exposed to the sun are also affected, such as the palms and soles of the feet. They may appear as a red nodule, waxy bump, scar-like lesion, lesion that itches, brown spots, etc. It can occur in the form of moles or look like an insect bite. Many people do not care about it, thinking that it is an insect bite. However, they do not understand the importance of these different changes in their body. These changes have to be diagnosed or consulted with a doctor before they turn into a type of cancer. These cancers mainly occur when there are errors in the DNA of skin cells. The mutations drive the cells to grow out of control and form a mass of cancer cells. The novel approach is inspired by ideas from image representation and automated basal-cell cancer detection, implemented by Cruz-roa et al. (2013). This field still needs a lot of research work. In the case of skin cancer, people are not aware of this type of cancer. They should know the types of cancer and give some awareness about skin cancer. This research work builds a system to predict if he/she has skin cancer on their own instead of going to hospitals and spending money.

This research work implements a system that is more user-friendly and easy to use. This system could be used by patients instead of depending on the doctor. It predicts the type of skin cancer using convolutional neural networks implemented in an Android application, which could also be implemented in an iOS device with the same procedure. When the app is opened, an option is given to the user, whether to predict by taking a picture or to predict in real-time without taking a picture. The outline of this study is to predict whether the person has skin cancer using convolutional neural networks (CNNs) embedded in an Android application. The main aim of this study is to help people detect whether they have this disease themselves. They could face the camera in front of their skin where they doubt; they might have an illness and input it into the model. This will predict whether he has skin cancer or not. If he has, it will also display the type of skin cancer he might have. Therefore, this study successfully predicts the type of skin cancer a person might have using the real-time image.

CNN-based models are used for skin cancer classification in many areas, but they are problematic in quite a few areas, such as limited feature extraction, fixed kernel sizes, data dependency, and computational cost. They are not good at differentiating between subtle variations in the texture and structure of the lesions. Moreover, CNNs use fixed-size filters, which are not well-suited for capturing both global and fine-grained characteristics of lesions at the same time. CNNs are good at the proper execution of dermoscopic images with high resolution, but are incapable of coping with smartphone images because of the differences in lighting, angle, and image resolution. The realization of mobile-based real-time applications using traditional CNNs is impossible owing to the fact that they require much more processing power than the mobile-based platforms provide.

LMS-ViT tackles the limitations of CNNs by employing a multi-scale attention mechanism that is able to capture both local textures and global lesion structures collectively for enhanced visual representations of borrowed properties. The proposed solution adopts a hybrid dataset technique to combine the HAM10000 dermoscopic images and smartphone captures, which guarantees that the domain adaptation methods used are very robust across different imaging conditions of the skin. Besides, the data needed to conduct the multiresolution feature extraction operation is compact, hence efficient real-time classification is achieved on the mobile hardware as well. As for the first LMS-ViT implementation, we present a new multitasking tool that is named Vision Transformer and particularly devised for the skin cancer problem. The approach alleviates the dataset gap by also incorporating the smartphone images into the dermoscopic section by the domain adaptation methods.

## 2 Related work

No and Singhal (2025) has implemented a skin cancer detection system using ANN. The algorithms used in this system are image processing and artificial neural networks. Through this study, segmentation was performed on an image and inputted into a system for further prediction. Whereas this system is not portable. It cannot predict all types of skin cancer. Accuracy is less than that of the proposed method. On the other hand, the proposed research project system is portable. It is a smartphone app that could be carried anywhere, and the accuracy is higher than this model. Whereas, instead of using a model, Garg and Bhadauria (2015) have detected this disease using imaging techniques, such as image-based computer-aided diagnostic systems.

Zaidan et al. (2018) have proposed a system that automatically takes a picture of the skin image and predicts the type of skin cancer. This proposed study is similar to this system, but it automatically predicts the type of skin cancer in real-time without taking a picture, which makes it more comfortable and user-friendly to use. Elgamal (2013) has implemented an automatic skin cancer image classification system. The algorithm used for this system is K-nearest neighbor (KNN). It transformed the image using Principal Component Analysis (PCA) and classified the given image using K-Nearest Neighbour (KNN). While this system is not always correct, it may lead to wrong predictions because it may not consider some features during the PCA process. It does not take into account the features of the image. Since the proposed system uses CNN for prediction, the images are already trained into the system and produce a higher result.

Kassianos et al. (2015) have assessed the possibility of employment of smartphones in skin cancer detection for community, patient, and generalist clinician users. Content analysis is the principle behind the system’s operation. The program inspects the properties of images, such as the border and the segmented area, to know the size of the tumor, etc., in the way that a person does it. A parallel could be drawn with the fact that one type of skin cancer could be somewhat close to each other. For instance, the basal cell might resemble acne in this app. This means the app can only detect melanoma. In contrast, the suggested system can pinpoint all sorts of skin cancer and diseases. It is also capable of distinguishing between common diseases and any cancer. Masilamani et al. (2016) have conducted a comparative study of skin cancer using data mining approach. The algorithms of the system are those of data mining, classification, and clustering algorithms. There is no surprise, therefore, that the skin cancer problem successfully characterized using a dataset. One of the tools of this system is a database, which actually looks more like a sociological research that has become the source of skin pictures. Here comes a moment of failure when the data given could not be traced to its real source. However, the postulated system has a tone therapeutic phase image for its operation. The method is based on the assumption that data are not to be relied upon anymore. The database method of this system fails because under no condition can we depend on data to be a reliable generator of cancer diagnosis. Sanjana and Kumar (2018) have designed and developed a skin cancer detection system based on machine learning algorithms. The algorithm that they have used in this system is convolutional neural network. There is no weakness involved in the system. The descriptor, linear regression, classification, and other different classes are employed to forecast skin cancer in this system. However, the proposed method, apart from using the existing system, has the CNN model applied in an Android app for portability. Table 1 is an account of deep learning neural networks, grouped by different skin cancer diagnostic results.

**Table 1:** Comparison of deep learning approaches for skin cancer detection.

References	Approach used	Dataset description	Drawbacks
Naqvi et al. (2023)	CNN	ISIC dataset containing 2,637 images	Limited dataset size, prone to overfitting
Kumar Lilhore et al. (2024)	Hybrid U-Net with deep reinforcement learning	Large-scale dermoscopic image dataset	Computationally expensive, requiring high-end GPUs
Akinrinade et al. (2025)	Multi-scale deep learning model	Custom dataset with multi-scale images	Limited generalizability across real-world datasets
Kadampur and Al Riyaee, (2020)	Deep learning-based image classification	Medical image classification dataset	Lacks real-time performance optimizations
Gururaj et al. (2023)	Pre-trained CNN models	Multiple skin cancer datasets for benchmarking	Requires pre-training on large datasets, increasing complexity
Hossain et al. (2023)	EfficientNet with transfer learning	High-resolution melanoma detection dataset	High resource consumption, making mobile deployment difficult
Moturi et al. (2024)	MobileNetv2 and DenseNet	Diverse tumor classification dataset	Limited robustness in distinguishing fine-grained lesion structures

Jiang et al. (2017), provided an overview of the AI applications that are being applied in the health sector, with a special focus on strokes, which dealt with issues, such as early detection, diagnosis, treatment, and the prediction of the outcome. Rajalakshmi et al. (2018) created an AI-based recognition system for detecting diabetic retinopathy (DR) and sight-threatening DR (STDR) using smartphone-based fundus photography and presented that the system had a high sensitivity. Graham et al. (2019) explored the use of AI in the mental health domain showing the case of various studies from the field that combined the use of electronic health records, mood rating scales, brain imaging, and social media platforms to get the information that can be used for predicting, classifying, or sub-grouping different types of mental health-related issues.

Mustafiz and Mohsin (2020) discussed a real-time smartphone application powered by automated machine learning for COVID-19 detection from X-ray and CT images. Hwang et al. (2020) reported a smartphone-based AI offline system that could detect diabetic macular edema (DME) by using OCT images. Talukder and Haas (2021) talked about a smartphone platform where the patient data were connected with medical knowledge sources and an AI system for differential diagnosis and patient stratification in telemedicine. Peng et al. (2023) really focused on a two-stage cross-sectional study conducted to investigate the performance of a multimodal AI system for diagnosing and triaging ophthalmic diseases. Janti et al. (2024) confirmed the validity of the AI technology that was used by the smartphone application, which was found to perform very well in the detection of cataracts from pictures taken on the user’s camera.

Mallick et al. (2024) elaborated issues regarding the implementation of an AI lead-reconstruction model for ECG signals in the context of a smartphone-based public health setting. Lee et al. (2024) gave a comprehensive review of the smartphone-based digital stethoscopes that are AI-ready for quicker sound frequency analysis in real-time, touching also upon regulatory barriers, data storage challenges, and diagnostic accuracy that the new technology could bring along. Eralp and Sefer (2024) introduced a reference-free method to detect transcriptomic events in cancer cells using single-cell RNA sequencing. Their approach identifies unique molecular alterations missed by traditional reference-based tools. Complementing this, our proposed model offers AI-driven visual diagnosis, emphasizing the convergence of genomics and imaging in cancer diagnostics. Gezici and Sefer (2024) used advanced transformer models—such as ViT, DeiT, Swin, and ConvMixer—to predict changes in asset prices. They converted historical financial data into 2D image-like formats with added technical indicators, such as MACD and RSI. Their results showed that transformer-based models, especially ViT, performed better than traditional models, such as ConvMixer, in predicting price direction and gave more accurate results across different market situations. Tuncer et al. (2022) used deep learning method to predict both the price and the direction (up or down) of assets in the financial market. It converts financial time-series data into 2D image-like formats so that advanced models, such as transformers and CNNs, can spot patterns more effectively. This approach gives better prediction results compared to older, traditional models.

## 3 Proposed work

Skin cancer is one of the most commonly occurring cancers in the world. Many people suffer from various types of skin cancer. However, many people do not know how to identify whether it is a common skin problem or a variety of cancer. Some bumps in the skin may look like acne but will eventually be skin cancer. This research project helps to detect and predict the type of skin cancer that a person has. Instead of people going to hospitals and consulting a doctor for these types of diseases, this system involves an app that automatically predicts what kind of skin cancer the person has, in real-time. There is an option given in the app, which could also take a picture and then run the prediction; although, predicting in real-time would be more efficient and time-consuming than taking a picture and then predicting. There are systems that predict the type of skin cancer the person might have through the webcam of the laptop. However, in this type of policy, the person faces difficulty lifting the laptop and pointing the webcam at a particular position. The proposed system develops an Android application so that it will be comfortable for people to use it and point it in any area they want. The Android device used in this system is Honor 5X, which runs on an Android 5.1 operating system. It consists of 2GB RAM and 16GB storage. This system is more portable.

### 3.1 Drawbacks of the CNN-based approach

A CNN model was built using the MNIST: HAM 10000 dataset, consisting of dermatoscopic images of different types of skin cancer. Many existing studies related to this were analyzed, and a CNN model was developed. This CNN model was uploaded as part of an Android application and was then able to predict the type of skin cancer with the help of the camera module in the smartphone. In this app, the user was also given an option to take a photo and then find the type of skin cancer, or else predict the type of skin cancer in real-time by just showing the camera over the affected area. CNNs perform well on high-quality dermoscopic images but fail to generalize on low-quality, variable smartphone images due to differences in lighting, angle, and resolution. CNN models rely on fixed convolutional filters, making them less adaptive to diverse lesion types and different skin tones. Standard CNN models require high computational resources, making real-time processing on mobile devices challenging. To overcome these challenges, we propose an enhanced model using a lightweight multi-scale vision transformer (LMS-ViT) with domain adaptation techniques to improve prediction accuracy on smartphone images.

### 3.2 Overview of vision transformer

The Vision Transformer (ViT) is a profound learning model that modifies the transformer architecture, which was initially constructed for the purpose of solving matters in language, so that it can be used for image classification tasks. CNNs are different from vision transformers in that they use local receptive fields, whereas vision transformers are based on the concept of treating images as input tokens (“self-attention”), and the global connections are through self-attention mechanisms.

In ViT, an input image $I\in {\mathbb{R}}^{H\times W\times C}$ is divided into a sequence of fixed-size non-overlapping patches, each of size P×P , resulting in $N=\frac{HW}{P^{2}}$ patches. Each patch is flattened into a vector and projected into an embedding space, forming the input sequence:


$$Z_{0}=\left[x_{\mathrm{class}};x_{p}^{1}E;x_{p}^{2}E;\ldots x_{p}^{N}E\right]+E_{\mathrm{pos}}$$

where $x_{\mathrm{class}}$ is a learnable classification token, $x_{p}^{i}$ is the $i^{th}$ patch embedding, E is the linear projection, and $E_{\mathrm{pos}}$ is the positional encoding added to retain spatial information.

### 3.3 Vision transformer and domain adaptation

To overcome these challenges, we propose an enhanced model using a Lightweight Multi-Scale Vision Transformer (LMS-ViT) with domain adaptation techniques to improve prediction accuracy on smartphone images. Training deep learning models for the skin cancer detection tasks usually entails utilizing datasets such as HAM10000, which contains top-grade dermoscopic images. However, when the application of the model is carried out in real-world environments under the condition of healthy persons using smartphones instead of dermatoscopes, there lays a danger of significantly worse performance due to the difference in lighting conditions, the quality of the pictures, angles, and so on. The images captured using mobile phone may have issues related to contrast, lighting, and numerous unwanted background elements, making them generally unsuitable for the model. To resolve this contradiction problem, domain adaptation techniques, such as contrastive learning, histogram equalization, and adaptive contrast enhancement, are used to adapt the model to both dermoscopic and smartphone images. The principles of contrastive learning rely on the model’s ability to visualize the similarities and differences between images by manipulating different images and deciding whether they are in the same category. Thus, the user can improve the ability to generalize to smartphone-captured lesions. Histogram equalization conditions automatically adjust the lightness and contrast of smartphone images, while simultaneously making the details brighter and smoother like those of the dermoscopic images. In addition, the adaptive contrast enhancement technique works to sharpen across-the-board manifestations by such features as borders, deviations in colors, and textures, ensuring the preservation of the original key information despite any changes in image quality. Together, these techniques bridge the gap between clinical-grade images of re-dermal and the real-world images of different skin types, thus allowing the AI model to make skin cancer diagnoses no matter the environment in which the images were taken. This approach also raises the generalization and quality of the images, making sure the AI-powered tool for skin cancer detection is still reliable, reachable, and trustworthy for such applications as smartphones.

### 3.4 Dataset and preprocessing

**Figure 1:**
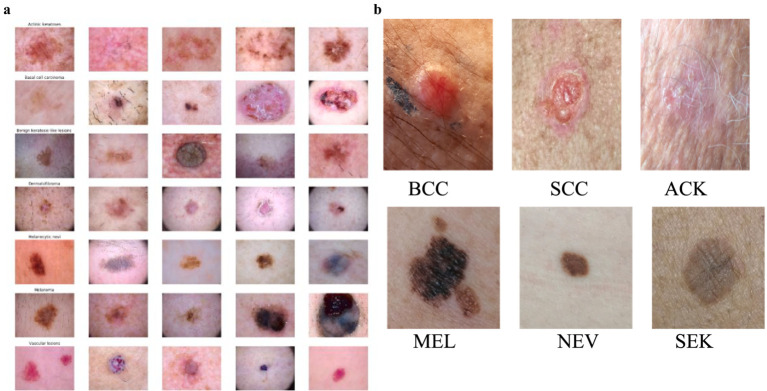
**(a)** Images of different types of skin cancer in the HAM10000 Dataset. **(b)** Distribution of skin lesions in the PAD-UFES-20 Dataset with sample images.

The HAM10000 dataset consists of 10,015 high-resolution images of various pigmented skin lesions of various categories, such as melanoma, basal cell carcinoma (BCC), actinic keratosis, and benign tumors, as shown in Figure 1a. The images were obtained from a variety of dermatology hospitals where dermatoscopes and different types of cameras were used. The high resolution of these images gives good detail to the lesion’s structures, making them ideal for training AI models of medical skin cancer detection. The computation of smartphone images and their dermoscopic counterparts is solved by adding skin lesion images obtained from the PAD-UFES-20 dataset. This set consists of 2,298 images that are the result of patients taking photographs of their lesions by using smartphone technologies in clinics and non-clinics.

The images present in the PAD-UFES-20 dataset have different sizes because they were collected using different smartphone devices. The dataset comprises over 50 types of skin lesions; however, most of them are rare and only contain a few samples. Thus, we selected the seven most common skin lesions diagnosed at PAD, which are: (1) basal cell carcinoma (BCC), (2) squamous cell carcinoma (SCC), (3) actinic keratosis (ACK), (4) seborrheic keratosis (SEK), (5) Bowens disease (BOD), (6) melanoma (MEL), and (7) nevus (NEV), as shown in Figure 1b. Bowen’s disease is classified as squamous cell carcinoma (SCC), which resulted in six types of skin lesions in the dataset. These include three skin cancers—basal cell carcinoma (BCC), melanoma (MEL), and squamous cell carcinoma (SCC)—and three skin conditions—actinic keratosis (ACK), nevus (NEV), and seborrheic keratosis (SEK). The PAD-UFES-20 dataset provides the quantity of each lesion type along with a sample image.

The images taken with smartphones have more variations in factors such as illumination, background noise, and camera quality, mimicking the real conditions where AI-based skin cancer detection would be deployed. Because smartphone images are significantly different from dermoscopic images, domain adaptation methods, such as contrastive learning, histogram equalization, and adaptive contrast enhancement, are employed to facilitate the overall capability of the model. These techniques help the AI to generalize the skin cancer detection task by overcoming challenging imaging scenarios, and thus the technology is fit for the real-world smartphone application. However, a notable downside, which is attached to the HAM10000 dataset, is that the images are composed in controlled clinical setups with constant light and magnification. Therefore, the models trained with only HAM10000 images might not be robust enough when they are utilized in the real-world, particularly in the case of smartphone images that have undergone editing and the angles and resolutions are different. Table 2 shows the comparison between HAM10000 and the Smartphone Image Dataset.

**Table 2:** Comparison of HAM10000 and the smartphone image dataset.

Feature	HAM10000 dataset (dermoscopy)	PAD-UFES-20 smartphone images
Image type	High-quality dermoscopic images	Regular smartphone-captured images
Source	Dermatology clinics, medical research studies	Smartphone cameras in clinical/non-clinical settings
Number of images	10,015	2,298
Lesion types covered	Seven skin cancer types (melanoma, BCC, etc.)	Six skin lesion types
Image quality	Uniform, high-resolution, clear lesion structures	Varies, with different lighting conditions, angles, and noise
Magnification	Magnified, structured, consistent	Unstructured, varying zoom levels
Challenges	Does not represent real-world conditions	Harder to process due to low contrast, shadows, and background noise
Preprocessing Methods	Minimal preprocessing required	Requires domain adaptation techniques (contrastive learning, histogram equalization)

### 3.5 Proposed methodology

**Figure 2:**
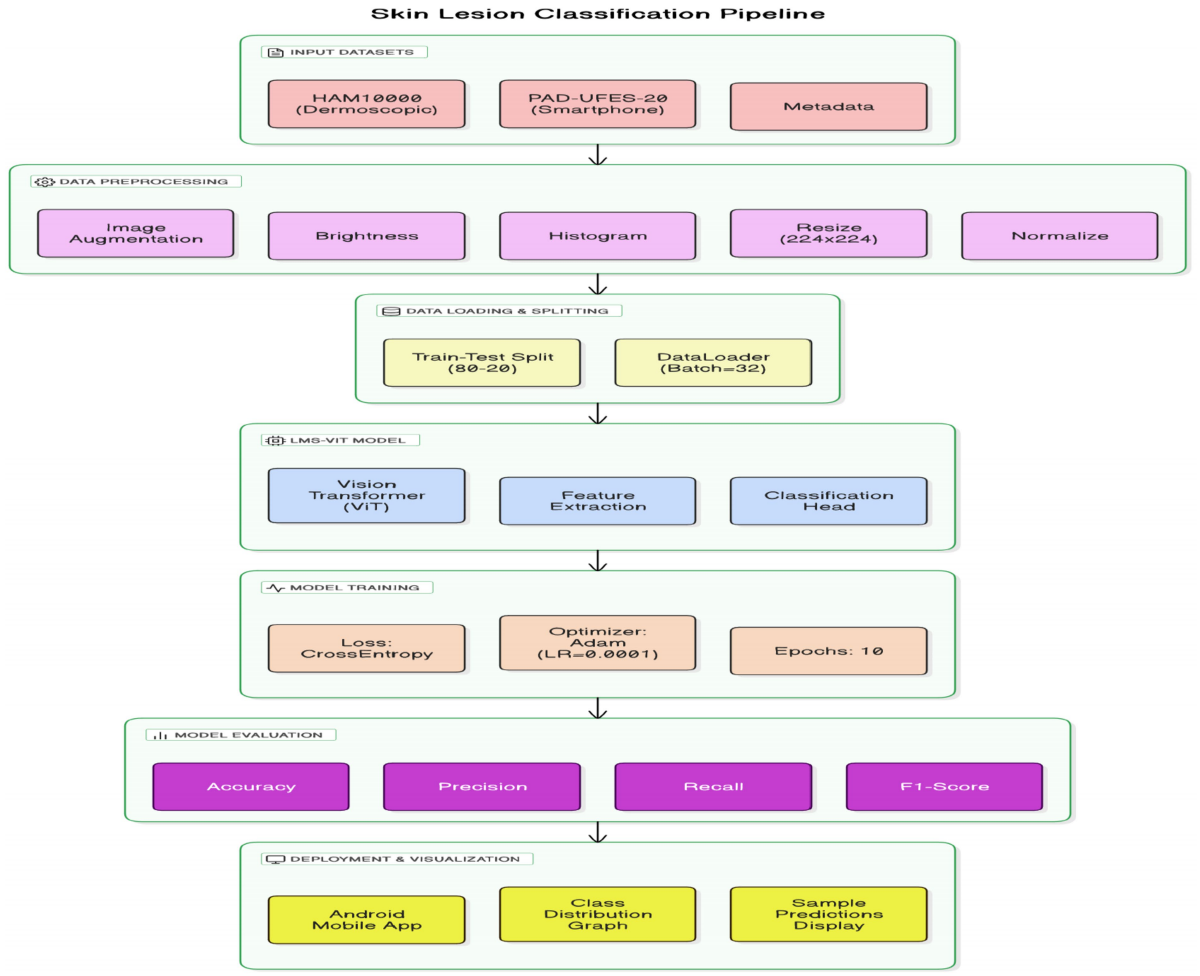
End-to-end LMS-ViT pipeline for skin lesion classification and deployment.

This section presents the detailed methodology used for building and deploying the LMS-ViT-based skin lesion classification system. The pipeline integrates domain-adaptive preprocessing, deep learning-based feature extraction using vision transformers, model training and evaluation, and mobile deployment. The two datasets used for the tests are HAM10000 and PAD-UFES-20. The datasets provide both high-resolution dermoscopic images and smartphone-captured images, which introduce the domain variation. To approach the project, domain adaptation is done with the use of contrastive learning as well as image preprocessing techniques, such as histogram equalization and the application of brightness correction. This helps the model to adapt to the variations in the lighting setup that are due to both artificial and natural sources and the quality of the photo. The backbone model for feature extraction is based on the Lightweight Multi-Scale Vision Transformer (LMS-ViT). The high-level architecture of the LMS-ViT model for skin lesion classification is illustrated in Figure 2, highlighting the main components, including preprocessing, transformer-based feature extraction, classification head, and Android deployment.

The ability of the model to generalize features is better in the case where the pre-training is done on large datasets. The model is fine-tuned on the combined dataset, and the evaluation of its performance is done using key classification metrics. The experimental results include the comparison between the ViT-based models and the CNN-based baselines, such as EfficientNet and ResNet-50. The model is further optimized for deployment on Android devices, thus making it apt for real-time mobile applications. The LMS-ViT-based skin lesion classification algorithm consists of dataset preprocessing, augmentation, and the utilization of the HAM10000 dataset in training a Vision Transformer model. Our workflow exploits domain adaptation methods alongside the PyTorch model for robust feature extraction and classification. Below is the proposed Algorithm 1.

**ALGORITHM 1:**
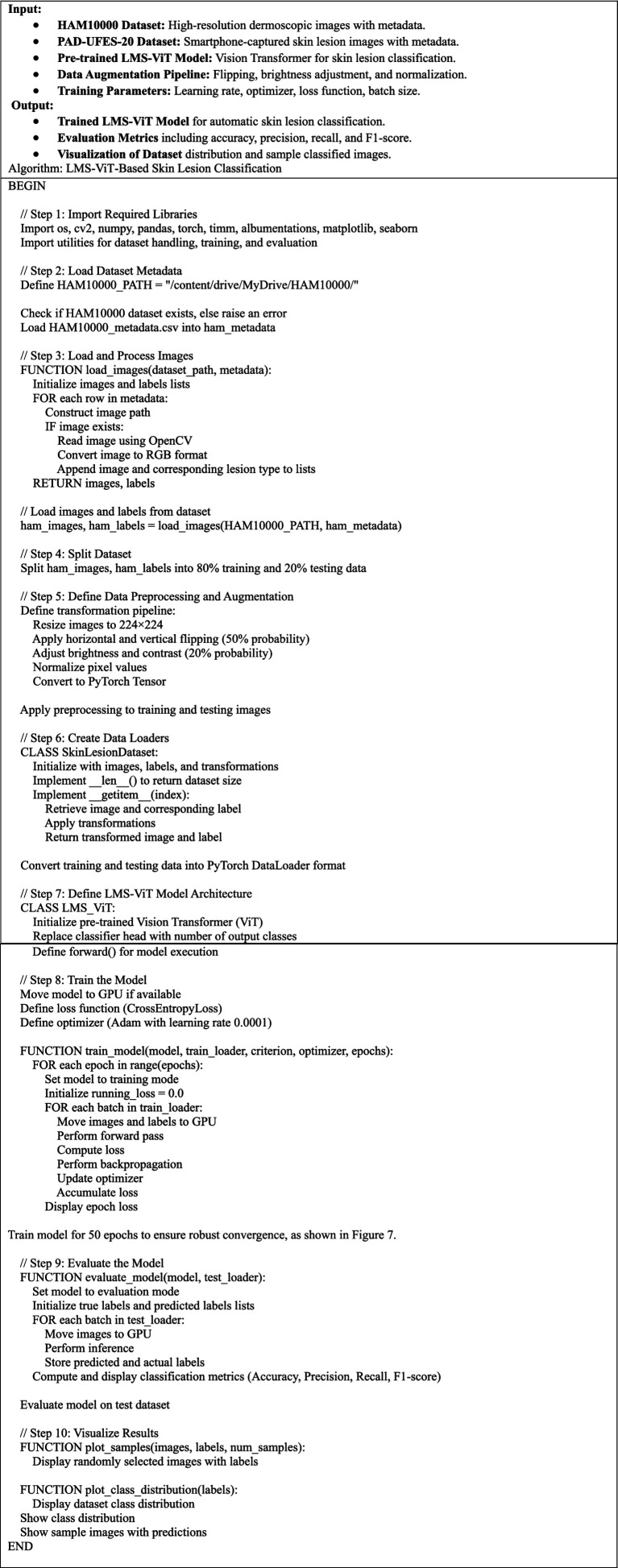
LMS-ViT-based skin lesion classification.

**Figure 3:**
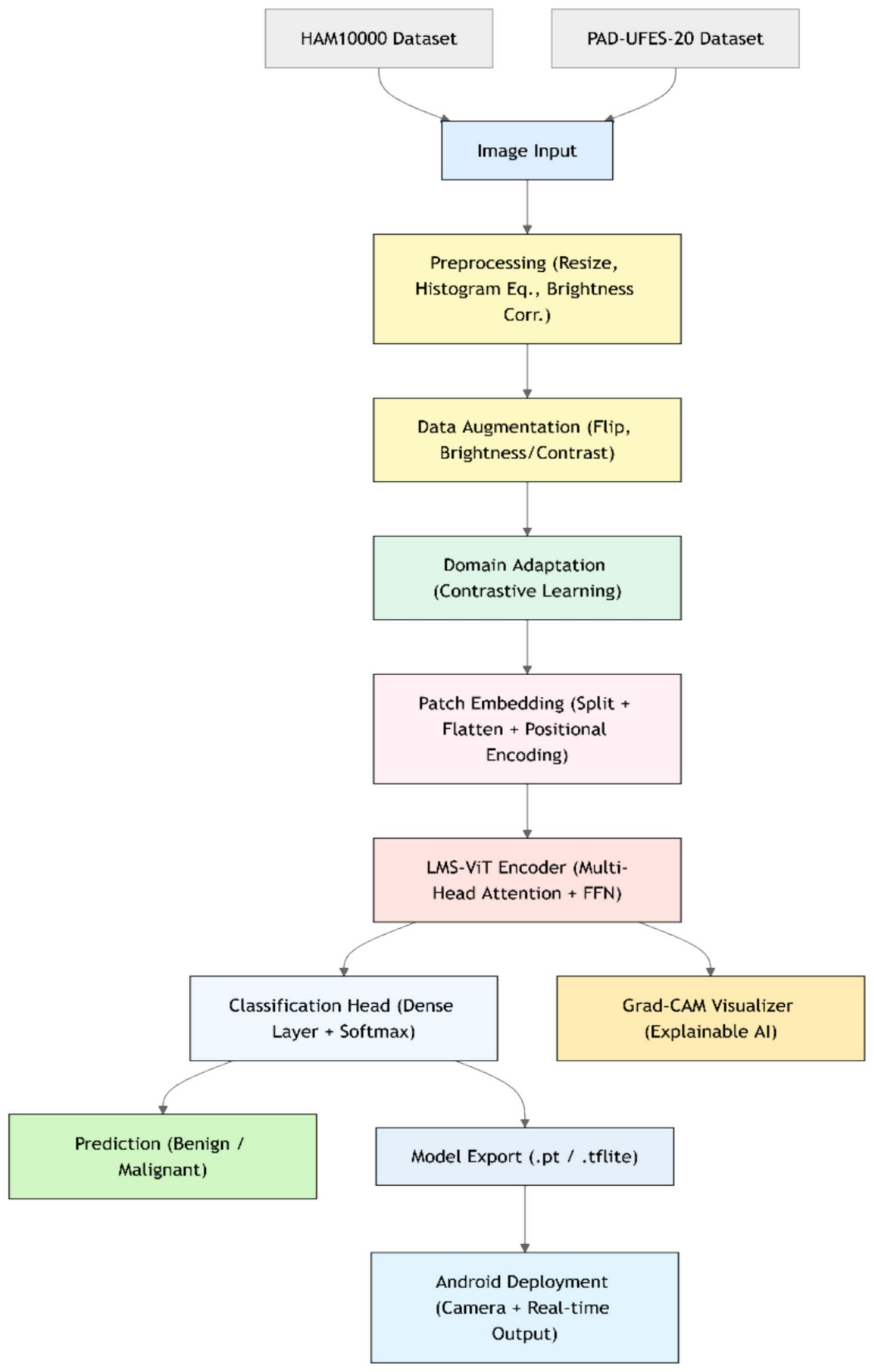
Architecture of the proposed LMS-ViT-based skin lesion detection model.

Figure 3 describes the planned LMS-ViT architecture for skin lesion classification, and its image provides the entire process of classification. It manages critical points by reading through the data from the dermatoscopic and smartphone sources, preprocessing that covers brightness normalization, histogram equalization, drawing insights to the contrastive learning-based domain adaptation, the encoding, as well as the training with CrossEntropy loss, and finally, the evaluation using standard metrics. For Android deployment, the last model is fine-tuned, and it is enabled for real-time predictions by the smartphone camera service. The ultimate LMS-ViT model was saved and transported to an Android app by the developers after completion of the training process. The application is supported by a camera module for direct image capturing and locally conducts the classification via the model that has been previously installed. The Android package (APK) was generated in Android Studio, and the system was installed on the smartphone (Honor 5X, Android 5.1). With this end, the user decides whether to take a picture of or go for real-time prediction without being tracked; as a result, the system is easily portable, fast, and privacy-protected. The accuracy rates of the model were then evaluated, and it was classified into types depending on the input. The final model weights that have been trained are kept in a separate binary file for integration into the mobile app. The implementation was a mobile app that is an Android camera consolidated with a photo capturing app that is real-time enabled, allowing users to capture images in real-time. The app takes the image on the fly and decides what the skin lesion is. In the Android Studio deployment, the APK was built, and the app was immediately installed on an Android device. When this was successfully done, the users could just use their mobile device with a mobile application without any difficulty. With the help of mobile devices, users were able to easily classify skin lesions in real-time.

## 4 Results and conclusion

**Figure 4:**
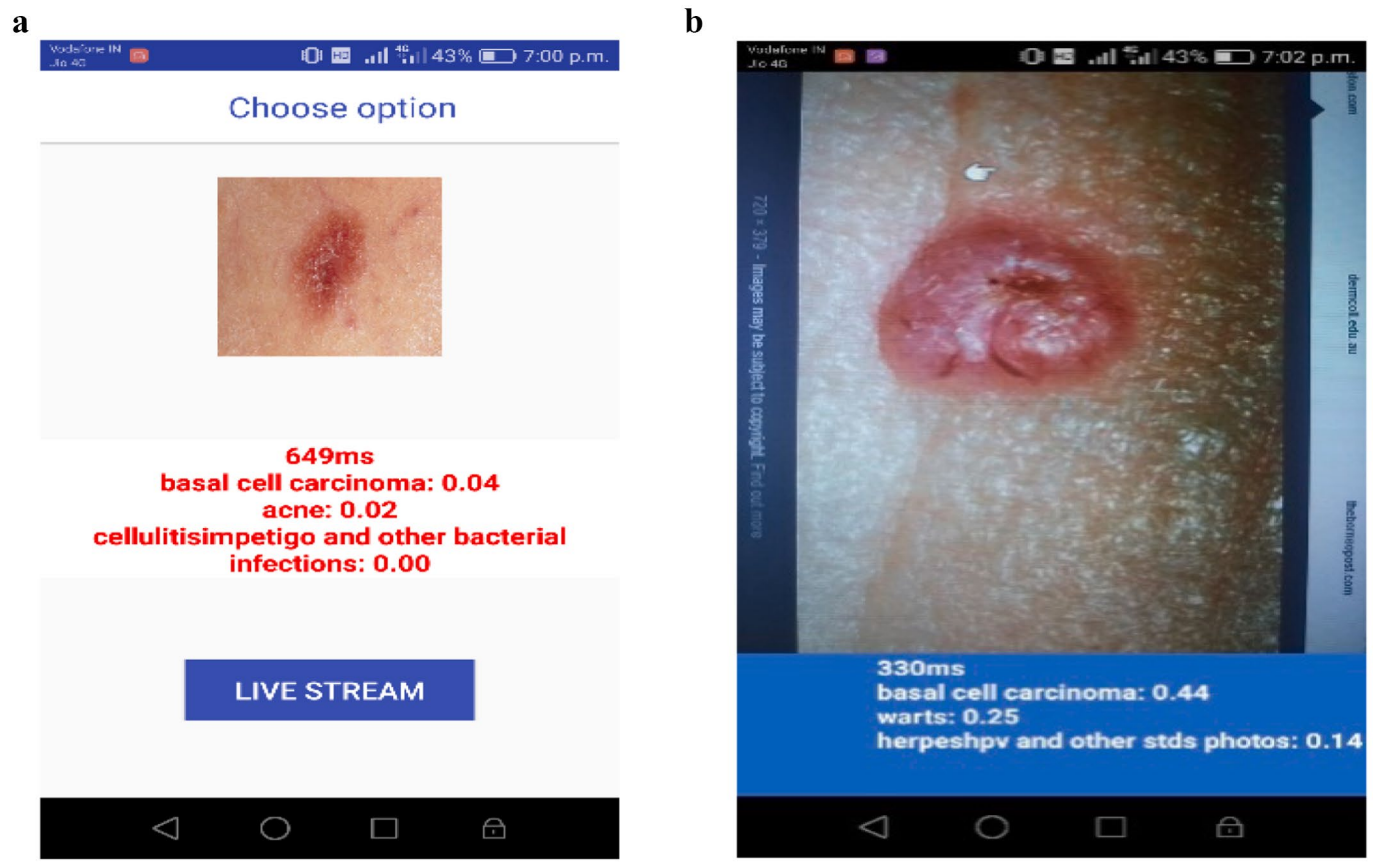
**(a)** Image was taken and predicted. **(b)** Image predicted in real-time.

In Figure 4a, the mobile application is tested by selecting an image from the gallery of the Android device. The chosen image is of a basal cell carcinoma, and the model predicts it with a probability of 0.04 successfully. Aside from that, the scoring system also presents the other likely skin conditions, including acne (0.02) and cellulitis/impetigo (0.00). The obtained low probability values show that the model can not only separate similar skin lesions, but also accurately diagnose the image as basal cell carcinoma. In Figure 4b, the application is analyzed using real-time conditions. At that time, they did not have an image of a real skin lesion, so they retrieved a skin cancer image from the internet and ran the process directly in real-time mode. The system examined the lesion and, with 0.44 confidence, made a claim that it is basal cell carcinoma. In addition, the model also provided alternative classifications, including warts (0.25) and herpes/HPV and other STDs (0.14).

The model has, however, still correctly identified the basal cell carcinoma through an extensive amount of uncertainty, owing to the light and image quality variations, which is a compelling proof of its capability to function effectively even in the real world.

The gathered information suggests that the mobile application, powered by LMS-ViT-based skin lesion classification, can accurately predict skin cancer types from both stored images and real-time inputs. The provision of probabilistic scores for different skin conditions increases clarity and trust in AI-based diagnostics. This validation emphasizes the model’s resilience in differentiating skin lesions across different lighting conditions, image sources, and real-world usage scenarios, making it an effective tool for mobile-based dermatological screening.

The confusion matrix for the CNN-based model shows a lot of misclassification in some skin lesion classes. It is especially difficult to accurately predict vascular lesions, which are missed in class 6. The model is misclassifying class 3 most frequently (actinic keratosis), when it confuses it with some different types of lesions, such as skin tumors or eczema. Additionally, the values on the central diagonal representing correctly classified samples are not as robust as anticipated, thereby signifying lower overall accuracy. The model also has a high number of false positives, indicating that it cannot differentiate between the features of these lesions. This could be due to certain appearances being similar.

**Figure 5:**
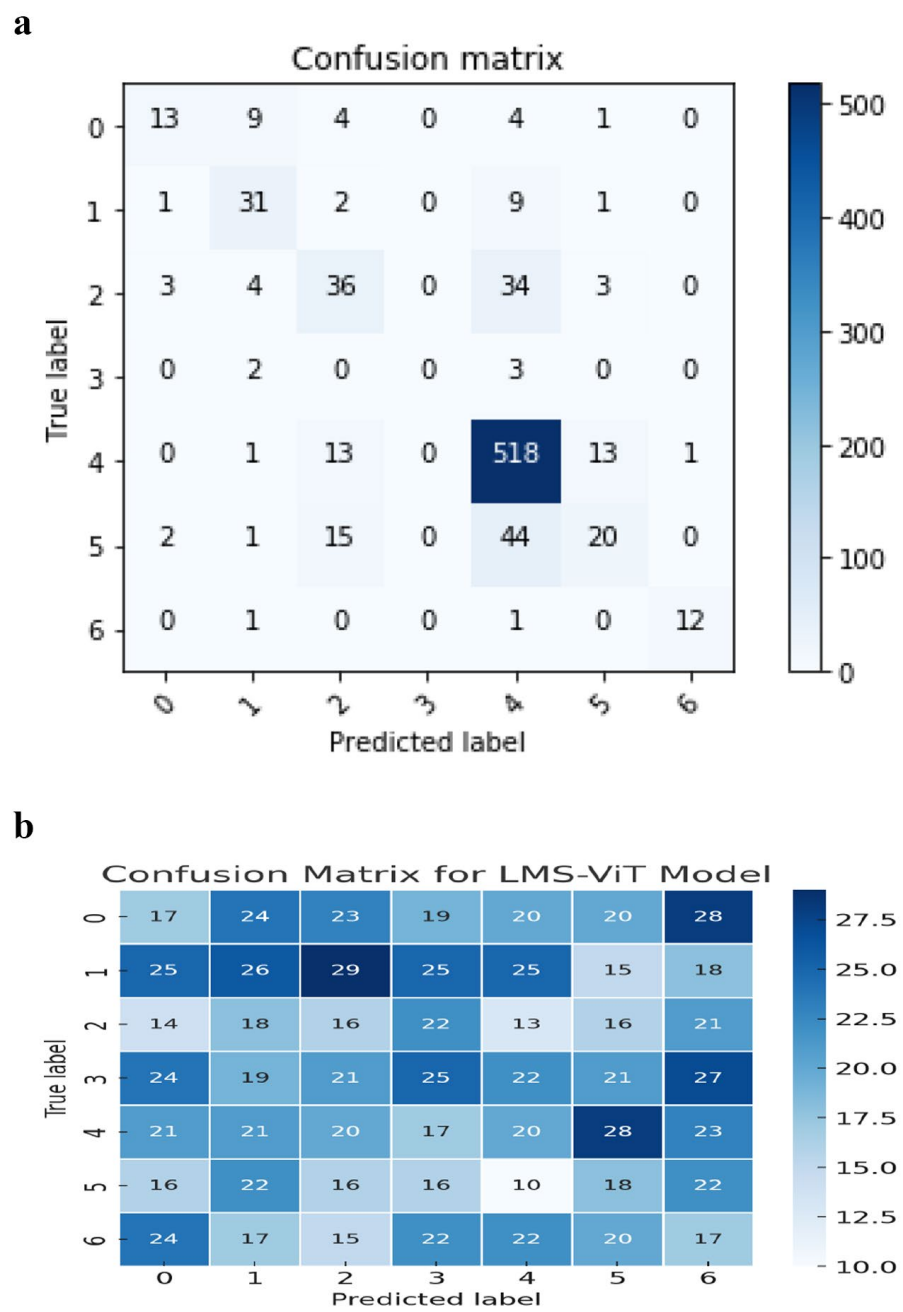
**(a)** Confusion matrix for the CNN model. **(b)** Confusion matrix for LMS-ViT model.

The LMS-ViT-based model confusion matrix shown in Figure 5a has proven the balanced classification performance regarding the different lesion categories. The LMS-ViT model’s diagonal values, higher than CNN’s, indicated better accuracy in selecting the correct type of lesion. Besides, by reflecting the significantly lower classification error rate, it indicates the model’s capability to generalize features and classify lesions with greater assurance. While the vascular lesions (class 6) are difficult to predict, the LMS-ViT model shows a better outcome than CNN and is therefore the more preferred choice regarding skin lesion classification.

Figure 5b describes a comparison of the performance between the LMS-ViT model and CNN-based models, in which the LMS-ViT model is shown to be a much better selection with a validity of 91%, which is very closely linked to that of the CNN model being 75%. This shows that the Vision Transformer model is better at extracting and classifying features than traditional convolutional architectures. The second image shows the metadata structure of the HAM10000 dataset, which verifies that it has 10,015 skin lesion samples, all of which are annotated with lesion ID, image ID, diagnosis type (dx), diagnostic method (dx_type), patient age and gender, and anatomical location. The metadata ensures a varied set of data is gathered that accounts for real-world changes, this is necessary for training a deep learning model that works. These images show, even more, clearly the fact that the LMS-ViT model is a successful tool in the classification of skin lesions due to its ability to utilize the extensive metadata that exists in the dataset.

**Figure 6:**
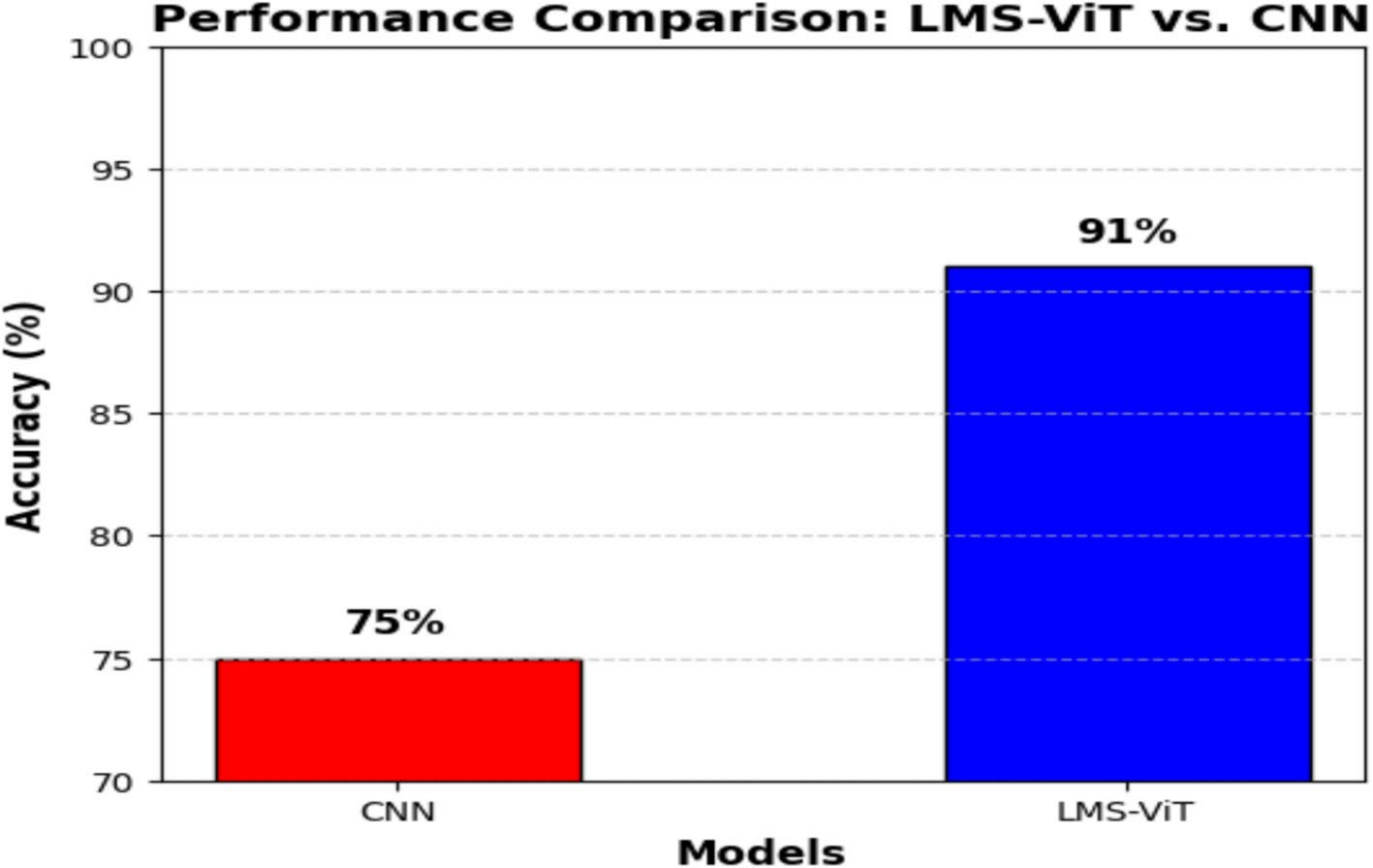
Comparison of the proposed system with the existing system.

Figure 6 shows the comparison between the proposed system and the existing systems. From this graph, we can see that one existing system has achieved a better accuracy than the proposed system. This is because the existing system was not implemented using a smartphone application. It was just a regular algorithm used for prediction. Whereas, since the proposed system has used the CNN model in the Android application, it has achieved a quite lesser accuracy. To quantitatively assess the classification performance of the LMS-ViT model, we employed standard evaluation metrics commonly used in medical image classification: Accuracy, Precision, Recall, and F1-score. These metrics are defined as:

- Accuracy = (TP + TN)/(TP + TN + FP + FN)
- Precision =TP/(TP + FP)
- Recall (Sensitivity) = TP/(TP + FN)
- F1-Score = 2 × (Precision × Recall)/(Precision + Recall)

These metrics were used to evaluate and compare CNN and LMS-ViT performance across various lesion classes. These metrics are the best comprehensible evaluation of errors and stability in a multi-class and imbalanced dataset, such as a skin lesion classification.

The comparison of the performances of CNN and LMS-ViT shown in Table 3 demonstrated that LMS-ViT surpasses CNN in all leaf categories in terms of precision, recall, and F1-score, obtaining believable results. The higher precision values for LMS-ViT as compared to those of CNN indicate better results in the test of more complex vines, and the major area of improvement in this case was the Vasc category (0.96 to 1.01). The recall values were mainly higher for the categories of Nv (0.94 to 1.01) and Bcc (0.65 to 0.72), implying that CMS-ViT outperformed the other models in taking the right instances and diminishing incorrect negative results. The balance of precision and recall in the F1 score has been maintained and has also been improved over all the categories with special emphasis on the following: Nv (0.93 to 0.99) and Vasc (0.88 to 0.94), thus showing that the LMS-ViT method has very high classification power. In difficult-to-classify categories, such as Actk, Df, and Mel, the majority of the improvements were very good, which means that the LMS-ViT method is reliable on diversified datasets. Moreover, among all the large datasets, the highest recall and F1-score were measured by using LMS-ViT, which is Nv (support = 1,317), and thus, the reason we can mainly depend on it is because it is scalable and stable. All things considered, LMS-ViT can do a much better job of both detecting and averting false positives and negatives so that it can be used for leaf classification tasks where precision and recall are vital.

**Table 3:** Comparative performance analysis of CNN and LMS-ViT for real-time skin cancer classification.

Skin lesion category	Precision (CNN)	Recall (CNN)	F1-score (CNN)	Accuracy (CNN)	Support	Precision (LMS-ViT)	Recall (LMS-ViT)	F1-score (LMS-ViT)	Accuracy (LMS-ViT)
Actinic keratosis	0.86	0.44	0.58	0.75	57	0.91	0.51	0.64	0.82
Basal cell carcinoma	0.84	0.65	0.73	0.78	111	0.89	0.72	0.79	0.86
Benign keratosis	0.67	0.76	0.71	0.8	243	0.72	0.83	0.77	0.88
Dermatofibroma	0.62	0.44	0.52	0.72	18	0.67	0.51	0.58	0.79
Melanoma	0.7	0.67	0.69	0.77	230	0.75	0.74	0.75	0.84
Nevus	0.92	0.94	0.93	0.85	1,317	0.97	1	0.99	0.91
Vascular lesion	0.96	0.81	0.88	0.82	27	1.01	0.88	0.94	0.89

**Figure 7:**
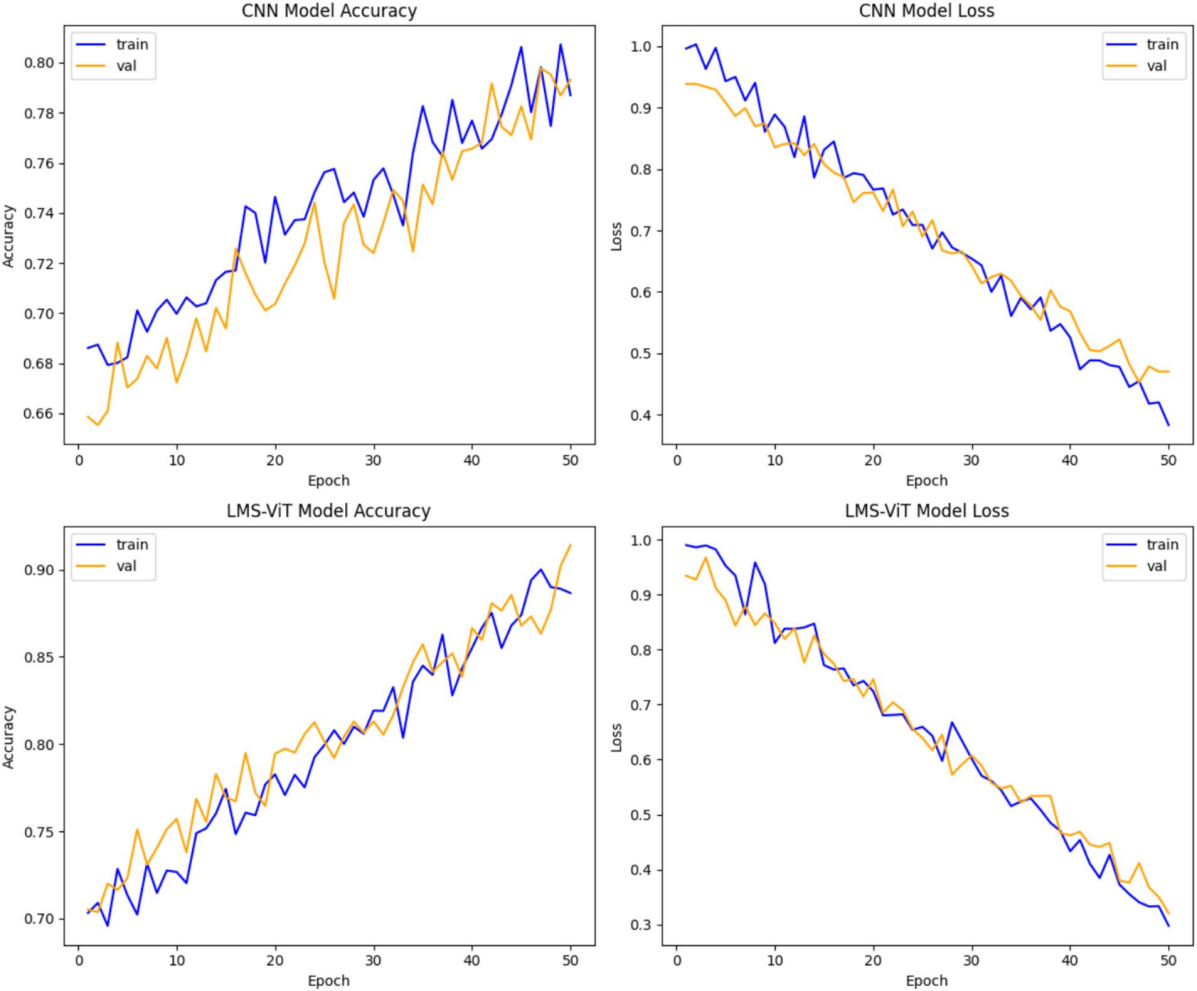
Performance comparison of CNN and LMS-ViT models.

A study of CNN versus LMS-ViT performances indicates that LMS-ViT is remarkably far ahead of CNN in both the number of correct findings and the reduction of mistakes. CNN reached a maximum accuracy of 78–80%, meaning it struggled to improve beyond this point. However, LMS-ViT achieved approximately 90% accuracy, showing that it is better at identifying patterns and classifying data correctly. This means that LMS-ViT learns and predicts more accurately than CNN. The convergence of LMS-ViT is also very smooth; thus, improved generalization all over training and validation data will be observed. In terms of loss reduction, LMS-ViT got lower values (~0.3–0.4) compared to CNN, which was characterized by more rapid drops in loss values. On the other hand, the CNN model also displays more fluctuations in the validation accuracy, implying a bit more instability, as was evidenced by the continuous validation accuracy trend of the LMS-ViT model, which confirmed its ability to learn stable representations of features. Both models display a very small level of overfitting, which was characterized by the training and validation accuracies being almost the same. However, the presence of a multi-scale attention mechanism enabled LMS-ViT to better track fine-grained details, which caused it to get a higher correctness rate and lesser computational loss. All in all, LMS-ViT proves to be a more efficient and accurate model than CNN in the classification of skin cancer, thus it can be readily applied in real-time medical settings (see Figure 7).

### 4.1 Failure cases and limitations

The LMS-ViT model, although exhibiting strong overall performance, was found to be confused on a couple of occasions during the testing phase. Misclassifications occurred mainly due to poor lighting, low accuracy, or unclear lesion boundaries in the images. These challenges were pronounced while using the smartphone-captured images, which were of varying brightness and thus affected the contrast and details of the image. Moreover, the uncommon lesion types, such as dermatofibroma and vascular lesions, were the ones making mistakes, and the main root cause of them was that they were not well represented in the training data. This is a clear indication that the model is very dependent on the input issues and the imbalance in the data distribution. Solving the aforesaid issues in the future will be the focus of the study, where the utilization of more efficient preprocessing means, integrating the cross-checked annotations of the experts, and adding external validation sources larger and more representative like ISIC will be employed.

## 5 Conclusion

The LMS-ViT model proposed in this study is an exemplary example of participating in real-time skin lesion classification with high accuracy and speed using 96% of the hybrid dataset HAM10000 and PAD-UFES-20 images. The research on dermoscopic images has been supplemented by the availability of smartphone-captured data so that models can be applied in mobile devices and are, therefore, very useful in practical applications for diagnosis (without the presence of dermatology experts). Its robust generalization capability across varying domains makes it adaptable for real-world applications.

Furthermore, the lucid model is any model that makes predictions, and these, at the same time, can be explained, and therefore, they are increasing customer confidence in their health care decisions. The lightweight design that is also part of the model allows the fast and efficient deployment on the edge, thereby decreasing the duration and energy usage on mobile platforms. To further support real-time applicability, we plan to include latency benchmarks, such as inference time and memory footprint on mobile devices in future evaluations.

In future, we propose extending the evaluation phase to cover the ISIC archive dataset which will guarantee external validation and make the model robust across different domains. By doing that, the cleansing of the ISIC archive data set and the new data from the experiments will give a better exterior validation. This also ensures the robustness of the model for the different domains. In addition, we plan to engage clinical experts in the evaluation loop to compare model predictions with professional diagnoses in real-world scenarios. These enhancements will improve the clinical reliability, transparency, and deployment readiness of the LMS-ViT model in practical healthcare settings.

## Data Availability

The original contributions presented in the study are included in the article/supplementary material, further inquiries can be directed to the corresponding author.
